# Smartphone App (2kmFIT-App) for Measuring Cardiorespiratory Fitness: Validity and Reliability Study

**DOI:** 10.2196/14864

**Published:** 2021-01-08

**Authors:** Adria Muntaner-Mas, Antonio Martinez-Nicolas, Alberto Quesada, Cristina Cadenas-Sanchez, Francisco B Ortega

**Affiliations:** 1 Department of Physical Education and Sports Faculty of Education University of Balearic Islands Palma Spain; 2 PROmoting FITness and Health Through Physical Activity Research Group Department of Physical Education and Sports, Faculty of Sport Sciences University of Granada Granada Spain; 3 Chronobiology Research Group Department of Physiology, Faculty of Biology University of Murcia Murcia Spain; 4 Ciber Fragilidad y Envejecimiento Saludable Madrid Spain; 5 Department of Biosciences and Nutrition Karolinska Institutet Stockholm Sweden

**Keywords:** exercise test, mobile apps, reproducibility of results, physical fitness, telemedicine, cardiorespiratory fitness

## Abstract

**Background:**

There is strong evidence suggesting that higher levels of cardiorespiratory fitness (CRF) are associated with a healthier metabolic profile, and that CRF can serve as a powerful predictor of morbidity and mortality. In this context, a smartphone app based on the 2-km walk test (UKK test) would provide the possibility to assess CRF remotely in individuals geographically distributed around a country or continent, and even between continents, with minimal equipment and low costs.

**Objective:**

The overall aim of this study was to evaluate the validity and reliability of 2kmFIT-App developed for Android and iOS mobile operating systems to estimate maximum oxygen consumption (VO2max) as an indicator of CRF. The specific aims of the study were to determine the validity of 2kmFIT-App to track distance and calculate heart rate (HR).

**Methods:**

Twenty participants were included for field-testing validation and reliability analysis. The participants completed the UKK test twice using 2kmFIT-App. Distance and HR were measured with the app as well as with accurate methods, and VO2max was estimated using the UKK test equation.

**Results:**

The validity results showed the following mean differences (app minus criterion): distance (–70.40, SD 51.47 meters), time (–0.59, SD 0.45 minutes), HR (–16.75, SD 9.96 beats/minute), and VO2max (3.59, SD 2.01 ml/kg/min). There was moderate validity found for HR (intraclass correlation coefficient [ICC] 0.731, 95% CI –0.211 to 0.942) and good validity found for VO2max (ICC 0.878, 95% CI –0.125 to 0.972). The reliability results showed the following mean differences (retest minus test): app distance (25.99, SD 43.21 meters), app time (–0.15, SD 0.94 seconds), pace (–0.18, SD 0.33 min/km), app HR (–4.5, 13.44 beats/minute), and app VO2max (0.92, SD 3.04 ml/kg/min). There was good reliability for app HR (ICC 0.897, 95% CI 0.742-0.959) and excellent validity for app VO2max (ICC 0.932, 95% CI 0.830-0.973). All of these findings were observed when using the app with an Android operating system, whereas validity was poor when the app was used with iOS.

**Conclusions:**

This study shows that 2kmFIT-App is a new, scientifically valid and reliable tool able to objectively and remotely estimate CRF, HR, and distance with an Android but not iOS mobile operating system. However, certain limitations such as the time required by 2kmFIT-App to calculate HR or the temperature environment should be considered when using the app.

## Introduction

Cardiorespiratory fitness (CRF) indicates the global cardiovascular, respiratory, and musculoskeletal capacity required to perform prolonged exercise [1]. There is strong evidence suggesting that higher levels of CRF are associated with a healthier metabolic profile [2-4], and that CRF is a powerful predictor of morbidity and mortality [1,5,6] Given the well-known relevance of CRF to general health status, its assessment has been strongly recommended in the recent American Heart Association scientific statement, proposing the assessment of CRF as a clinical vital sign [7]. Additionally, CRF assessment is important for testing the success of an intervention and for monitoring purposes.

The maximal oxygen consumption (VO_2_max) is an objective measure of CRF and has been considered to be its best indicator [8]. The American College of Sports Medicine provides guidelines, including different methods for CRF testing and ergo spirometry conducted during incremental maximal exercise tests on treadmills or a cycle ergometer, as these methods are considered the gold-standard measures of CRF [9]. In this context, the 2-km walk test (UKK test) is a widely used field-based battery of fitness tests such as the adult version of the EUROFIT battery and more recently the ALPHA fitness test battery for adults [10]. The UKK test has a distinct characteristic from most other field-based tests aiming to indirectly estimate VO_2_max (eg, the 6-min walk test). After completing the exertion (ie, walking 2 km as fast as possible), the tester records the time spent (which for a given distance is a measure of performance as an indicator of walk speed) plus the physiological response to that exertion (ie, the heart rate [HR]). If either of these two parameters changes, the estimation of VO_2_max would also change, making the test very sensitive to detect small changes in the CRF level, which is important for assessing the effectiveness of an intervention for monitoring purposes. Despite these advantages of the UKK test for measuring CRF, to properly perform this test, a valid and reliable HR monitor, stopwatch, and instrument (eg, measuring tape or GPS) are required to measure a 2-km route. It would be very useful and practical if a valid and reliable smartphone app could replace all of these instruments. Moreover, if a sports specialist, clinician, or researcher wants to assess the CRF level of several individuals, the individuals requiring the test need to visit the assessment center, which imposes a geographic limit as to who can be tested.

In this context, an app based on the UKK test would provide the possibility to assess CRF remotely for individuals who are geographically distributed around a country or continent, or even among those living on different continents, with minimal equipment and costs. The app would require the calculation of distance traveled, time, and HR at the end of the walk. To our knowledge, there is no currently validated app to estimate VO_2_max through the equations provided by the developers of the UKK test [11]. We have also not found any fitness app that integrates a measure of fitness performance (ie, walking speed estimated from measuring a 2-km distance and the time spent to complete it) and the HR measured into an estimation of VO_2_max using the camera of a mobile phone, which would remove the need for an additional monitoring device (eg, HR chest band).

Notably, some authors have demonstrated the validity of apps for measuring distance or HR in isolation. For instance, Benson et al [12] and Gordon et al [13] demonstrated the validity of a GPS-enabled iPhone app to track exercise distance. In addition, Martinez-Nicolas et al [14] revised the Runkeeper app and suggested its suitability for tracking distance. Otherwise, over the last few years, smartphone apps have gained the ability to measure HR by detecting the pulse using photoplethysmographic (PPG) imaging [15]. In this sense, Mitchell et al [16] examined the accuracy of Instant Heart Rate (Azumio) for pulse rate measurement, which was compared to that of an FT7 Polar HR monitor. The app was proven to be valid and reliable at rest and immediately postexercise. Likewise, Poh and Poh [17] showed strong agreement between HR assessments obtained using the Cardiio app against a Food and Drug Administration–eared pulse oximeter at rest and after moderate to vigorous exercise. Based on this background, we decided to develop an app ad hoc, named 2kmFIT-App, that can unite the measure of the distance walked, particularly the 2 km of the UKK test, the time needed to complete the 2-km walk, and the HR at the end of the test using the phone camera.

The overall aim of this study was to evaluate the validity and reliability of 2kmFIT-App developed ad hoc for Android and iOS mobile operating systems to estimate VO_2_max, in comparison with VO_2_max calculated following the original instructions and instruments of the UKK test. The specific aims of the study were to determine the validity of 2kmFIT-App to track distance and calculate HR versus a measuring wheel and HR monitor, respectively.

## Methods

### Development of 2kmFIT-App

**Figure 1:**
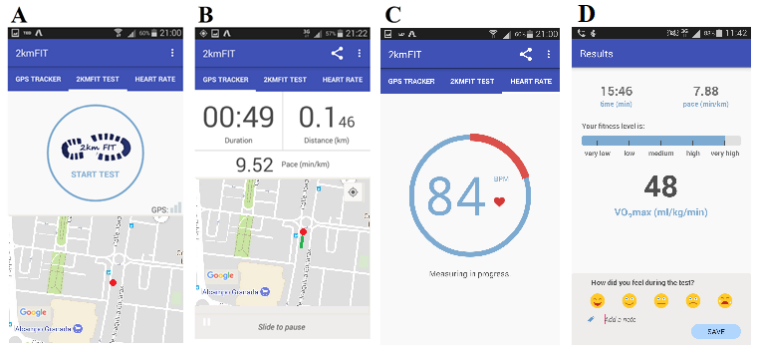
Main screens of the 2kmFIT-App Android version.

We developed 2kmFIT-App (Figure 1), which is able to track distance through the GPS of the smartphone, time, and HR using PPG imaging from the phone camera. The app was created to run on both major mobile operating systems. Android Studio 2.2.3 and Java 1.8_112 were used to develop the Android app, and the ButterKnife, Timber, AppIntro, DBFlow, Saripaar, Google Maps, CardView, and MPAndroidChart frameworks (Android Inc, USA) were used to design the required functionalities of the Android app. Likewise, Xcode 8.3.3 and Swift 3 were used for the Mac operating system to develop the iOS app, and the AVFoundation, MapKit, QuartzCore, Charts, Realm, and PermissionScope frameworks were utilized to create the required functionalities of the iOS app.

Different versions of 2kmFIT-App were developed, both for Android (versions 1 and 2) and iOS (versions 1, 2, and 3), aiming to enhance precision in measurements. Finally, the app was programmed with the equations of Oja et al [18] based on the original UKK test and to estimate the VO_2_max in both mobile operating systems. The CRF reference values of Rodriguez et al [19] were also programmed into the app. 2kmFIT-App has been registered in Intellectual Property Registry (Safe Creative register number 1904040536262). More detailed information on the structure and content of 2kmFIT-App can be found in Multimedia Appendix 1.

### Validation Protocol

#### Design

The validation evaluation of 2kmFIT-App was performed in two phases: in-laboratory validation (phase 1) and field-test validation and reliability (phase 2). A Samsung Galaxy SIII Neo (Android 4.4.2) and an iPhone 6s plus (iOS 11.3) were used for testing 2kmFIT-App during the two phases on the Android and iOS mobile operating system, respectively.

#### In-Laboratory Validation (Phase 1)

During this preliminary validation phase, different HR measurements were taken with 2kmFIT-App using the Android (version 1 and 2) and iOS (version 1, 2, and 3) versions, at two different exercise intensities (rest and moderate intensity). The HR measurement was tested because it is the most novel and challenging smartphone feature integrated into the app. In addition, during the UKK test, HR is taken only when the participant reaches the end of the 2-km walk. The objective of this first phase was to ensure that 2kmFIT-App could measure HR at the end of the test with a reasonable margin of error*.* One member of the research group examined the accuracy of 2kmFIT-App for HR measurement using both mobile operating systems under three different conditions: at rest, and with moderate and high exercise intensities. Additionally, four commercial iOS-based HR apps available in the App Store market were also tested to gather information on the accuracy of existing HR apps and to compare the HR accuracy of our developed app (see Multimedia Appendix 1 for detailed information).

#### Field Test Validation and Reliability (Phase 2)

##### Operating System

The second phase examined the validity and reliability of 2kmFIT-App using only the Android mobile operating system (version 2) in field conditions with 20 study participants. The iOS version of 2kmFIT-App was determined to have poor accuracy for HR measurement at rest and at medium intensity, and was therefore not included in phase 2. Accordingly, all findings presented herein refer only to the app running on the Android operating system.

##### Study Design and Participants

In this second phase, we used a cross-sectional design to test the validity of 2kmFIT-App by comparing the results obtained through the app data against criterion measures (UKK test). Additionally, we developed a test-retest design for testing its reliability in a repeated-measures analysis. A convenient sample of 20 healthy adults (25% female), who were mainly students from the University of Granada (Spain), were recruited for this study. We estimated the sample size needed for detecting correlation coefficients between the HR and VO_2_max assessed with the app and with the criterion methods equal to or higher than 0.7, with a standard α error of 5%. Our power calculation model showed that with 17 participants we would have 95% power to detect the expected correlations between methods. We finally included 20 participants to have some additional residual power. The study was approved by the Human Research Ethics Commission of the University of Granada (ref: 280/CEIH/2017) and abides by the bioethical principles set out by the Declaration of Helsinki. Participants received information about the characteristics of the study and data management. Participants also provided written informed consent to participate in the study. Data from the volunteers were included in a database and were protected according to Spanish Law 15/1999 of December 13, 1999.

##### Instruments

A professional TM12 measuring wheel from Top Measure was considered an accurate criterion measure for distance on the inner line of an athletic track. Criterion HR measurements were collected by an RS300X HR monitor from Polar (Kempele, Finland) and a smartphone was used as a stopwatch reference (LG G2 Mini, Android version 5.0.2). Weight (kg) and height (m) were obtained in one step using an electronic scale with an integrated stadiometer (Seca 769 scale with Seca 220 stadiometer, Hamburg, Germany) without shoes, in light clothing, and the Frankfort plane. BMI was calculated using the formula of weight/height^2^ (kg/m^2^). 2kmFIT-App was used during the performance of the UKK test as the instrument to be validated (see Multimedia Appendix 1 for a full description of the 2kmFIT-App structure and content).

##### Testing Protocol

The test was performed by the participant from 9 AM to 5 PM on an athletics track made of an artificial surface of polyurethane in an outdoor setting. The weather was mainly sunny, and temperatures ranged between 8°C and 26°C. All measures were collected from March 6 to 22, 2017 at the Faculty of Sports Sciences of the University of Granada (Granada, Spain). Participants performed the test twice (test-retest) with a 1-week interval between assessments. Before starting measurements, the experimental protocol was individually explained to participants, and then their weight and height were measured immediately afterward. The participants were then asked to wear the Polar RS300X watch and the chest strap. A user profile was created, and the name of the participant, along with their gender, birth date, weight, and height were entered into the “users” section of 2kmFIT-App.

##### HR Instructions

At the starting line of the test, participants were instructed on how to perform a valid HR measurement with 2kmFIT-App following the instructions shown in Textbox 1. Once the participants felt familiar with the process and obtained two valid HR measures with the app, they were considered to be sufficiently trained to perform the UKK test.

**Instructions for heart rate (HR) measurement using 2kmFIT-App.**

1. Stay standing, relax, and breathe normally.
2. Try not to swallow while measuring.
3. Place the index fingertip vertically on the camera.
4. Try not to move your finger, and apply constant pressure on the camera lens.
5. Do not apply excessive pressure.
6. Tap the button (with your other hand) on the screen to start the HR measurement and at this moment the camera’s flashlight will turn on.
7. Keep the phone stable until the HR measurement ends.
8. When finishing the walked distance, it is recommended to tap the camera lens with the finger as quickly as possible to reduce the time of HR recovery.
9. If the fingertip or ambient temperature is cold, the use of gloves during the walked distance is recommended.

##### UKK Test Instructions and Procedures

**Figure 2:**
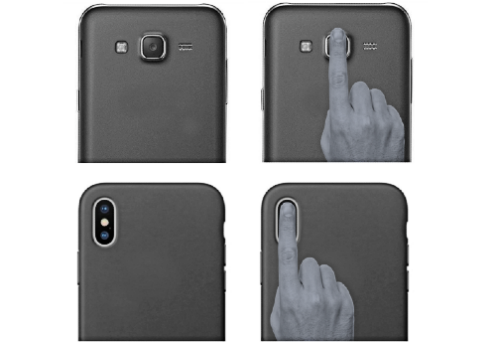
Correct placement of the index fingertip on different locations of the camera lens to obtain a correct heart rate measurement by photoplethysmographic imaging.

The tester reminded the participants of the instructions for performing the test following the original protocols and recommendations [18]. The app was then initialized (Figure 1A) and the UKK test was started. The distance was tracked using the app’s GPS feature (Figure 1B). When the participants approached the 2-km mark (1.8 km), the app emitted three beeps indicating that the test will soon be complete, and participants had to get ready for HR measurement using the smartphone camera. The app then indicated the end of the test with another audio signal. At this moment, the participants stopped walking and measured their HR using 2kmFIT-App, as detailed in the “HR instructions” section (Figure 2). Once 2kmFIT-App finished the HR measurement, the following outcomes were collected from the app: time spent to complete the test (ie, as 2 km estimated by the app), HR at the end of the test, and VO_2_max calculated by 2kmFIT-App. For the HR measurement, the tester also noted the outcomes obtained using criterion instruments on the provided datasheet. The outcomes were the three times recorded from the stopwatch (T0, T1, T2) and HR measured by the Polar monitor at three separate moments: P0, at the end of the walk (at the moment the final beep emitted by the app); P1, when the app started to measure HR; and P2, when the HR measurement was completed and shown in the app. 2kmFIT-App took an average of 25.85 (SD 0.98) seconds to obtain HR measurements. Finally, the criterion VO_2_max was calculated using the original equations of the UKK test and all of the criterion measures [11].

### Statistical Analysis

For the field-test validation and reliability (phase 2), the data of the UKK test from the 20 participants in total (2 trials) are presented as the mean (SD). A one-sample *t* test was used to evaluate whether the mean difference (ie, systematic error) between the app criterion measures was significantly different (*P*<.05) from zero (reference). The mean absolute percentage error (MAPE) was also calculated as the mean difference of the 2kmFIT*-*App outcome minus the respective criterion outcome (×100/mean criterion outcome). To complement this analysis, the intraclass correlation coefficient (ICC) was calculated between the criterion and 2kmFIT-App measurements, and between the test and rest. Interpretation of ICC values was based on standardized guidelines, in which a value less than 0.5 indicates poor reliability, values between 0.5 and 0.75 indicate moderate reliability, values between 0.75 and 0.9 indicate good reliability, and values greater than 0.90 indicate excellent reliability. Additionally, the Pearson product-moment correlation coefficient was calculated and linear regressions were performed to analyze the validity of 2kmFIT-App with criterion references and test-retest reliability. The agreement between the traditional UKK test and 2kmFIT-App measures was examined using the Bland and Altman method [20]. The mean difference (error) and 95% limits of agreement (error, 1.96 SD) were calculated. Results were graphically examined by plotting the differences against their mean. Bland-Altman analysis was performed with SigmaPlot 12.5 for Windows. Statistical analyses were performed using the Statistical Package for the Social Sciences software version 23.0 (SPSS Inc, Chicago, IL, USA). The level of statistical significance was set at *P*<.05.

## Results

### Participants’ Characteristics

Twenty participants were included in phase 2 for field-test validation and reliability analysis. Of note, 6 participants (from the test and retest) were excluded due to the following reasons: the participant failed to place the fingertip in the correct position when measuring (n=1), the smartphone did not provide data (n=2), or the smartphone froze during the test (n=3). Therefore, 16 men and 4 women ranging in age from 19 to 29 years (mean 24.96, SD 2.33 years) were included in the analysis. Descriptive characteristics of the participants are shown in Table 1, and their 2-km walk UKK test and retest results are shown in Table 2. During phase 1, only the Android version of 2kmFIT-App showed accuracy in HR measurement at medium intensity; therefore, no data are shown for iOS in phase 2 (see Multimedia Appendix 1 for these results).

**Table 1:** Characteristics of the sample (N=20).

Characteristic	Mean (SD)
Age (years)	24.96 (2.33)
Weight (kg)	69.73 (10.52)
Height (m)	1.74 (0.07)
BMI (kg/m^2^)	23.03 (2.29)

**Table 2:** Test and retest results of the 2-km walk UKK test (N=20).

Variable		Overall (test and retest), mean (SD)	Test, mean (SD)	Retest, mean (SD)	*P* value^a^
Outside temperature (°C)		15.80 (3.28)	18.40 (4.32)	13.20 (4.58)	.001
App distance^b^ (meters)		1942.60 (38.31)	1929.61 (51.47)	1955.60 (34.91)	.01
Pace (min/km)		8.29 (0.88)	8.37 (0.90)	8.20 (0.88)	.03
**Time (minutes)**					
	Criterion^c^	16.57 (1.75)	16.75 (1.79)	16.39 (1.77)	.03
	App^d^	16.09 (1.82)	16.16 (1.93)	16.02 (1.83)	.50
**Time (seconds)**					
	P0-P2^e^	25.85 (4.99)	25.40 (6.48)	26.30 (5.89)	.59
	P1-P2^f^	19.24 (4.95)	18.78 (5.87)	19.70 (6.34)	.57
**Heart rate (beats/minute)**					
	Criterion P0^g^	142.08 (17.37)	142.35 (16.54)	141.80 (19.48)	.81
	Criterion P1^h^	139.90 (17.66)	140.65 (16.77)	139.15 (19.92)	.53
	Criterion P2^i^	125.90 (19.78)	127.55 (16.84)	124.25 (23.89)	.23
	App^j^	123.35 (21.49)	125.60 (18.32)	121.10 (26.05)	.15
**VO_2_max^k^ (ml/min/kg)**					
	Criterion P0^l^	38.71 (5.17)	38.16 (5.27)	39.27 (5.20)	.007
	Criterion P1^l^	38.95 (5.25)	38.35 (5.29)	39.56 (5.35)	.004
	Criterion P2^l^	40.49 (5.71)	39.79 (5.73)	41.20 (5.87)	.006
	App^m^	42.21 (5.93)	41.75 (6.06)	42.67 (6.18)	.19

^a^Paired-samples *t* test between test and retest.

^b^Real distance at which the test measured by means of the app ends.

^c^Estimated time of the UKK test if mean walking speed up to 2 km as measured by the criterion method would have been maintained.

^d^Time taken to perform the test measured by the app.

^e^P0-P2: time difference between the final beep emitted for the app and showing the heart rate on the app screen.

^f^P1-P2: time difference between the start of heart rate measurement and the end of heart rate calculation by means of the app.

^g^Polar heart rate immediately when the test finished.

^h^Polar heart rate when starting the measurement with the app.

^i^Polar heart rate at the end of the measurement with the app.

^j^Heart rate measured by the app at the end of the test.

^k^VO_2_max: maximum oxygen consumption.

^l^Criterion VO_2_max P0, P1, and P2: VO_2_max estimated with the hypothetical arrival at the UKK test finish line considering walking speed and heart rate at P0, P1, and P2, respectively.

^l^VO_2_max estimated by the app calculation.

### Field Test Validation and Reliability (Phase 2)

#### Validity

Table 3 shows the mean differences among app minus criterion measures within the app, along with the ICC and MAPE values, which express accuracy as a percentage of the error. Of note, distance, time, and HR were underestimated by the app, and consequently overestimated VO_2_max. The systematic error was significantly different from zero for all studied outcomes (*P*<.001) except for HR at P2. In addition, there was moderate validity found for HR at P0 and good validity found for VO_2_max at P0 based on the ICC.

**Table 3:** Validity of 2km FIT-App (Android version) in comparison with criterion measures (UKK test).

Outcomes		2kmFIT-App measure, mean (SD)	Criterion measure (UKK test), mean (SD)	Difference (app – criterion), mean (SD)	*P* ^a^	MAPE^b^ (%)	ICC^c^ (95% CI)	*r* ^d^
Distance (meters)		1929.6 (51.47)	2000.0 (0.00)	–70.40 (51.47)	<.001	–3.52	N/A^e^	
Time (min)		16.16 (1.93)	16.75 (1.79)	–0.58 (0.44)	<.001	–3.58	0.971 (0.928-0.988)	0.97
**Heart rate (beats/minute)**								
	P2^f^ – P0^g^	N/A	142.35 (16.54)	–16.75 (9.96)	<.001	–11.77	0.731 (–0.211 to 0.928)	0.84
	P2 – P1^h^	N/A	140.65 (16.77)	–15.05 (9.56)	<.001	–10.70	0.770 (–0.197 to .0939)	0.86
	P2	125.60 (18.32)	127.55 (16.84)	–1.95 (7.83)	.28	–1.53	0.947 (0.869-0.979)	0.90
**VO_2_max^i^ (ml/min/kg)**								
	P2 – P0	N/A	38.16 (5.27)	3.59 (2.01)	<.001	9.41	0.878 (–0.125 to 0.972)	0.95
	P2 – P1	N/A	38.35 (5.29)	3.40 (2.03)	<.001	8.88	0.887 (–0.102 to 0.974)	0.94
	P2	41.75 (6.06)	39.79 (5.73)	1.96 (1.89)	<.001	4.93	0.948 (0.569-0.986)	0.95

^a^One-sample *t* test: the intertrial difference was entered as a dependent variable; the *P* value indicates whether the mean difference is significantly different from 0 for all measures.

^b^MAPE: mean absolute percentage error; calculated as ([study outcome – criterion measure]/criterion measure) × 100.

^c^ICC: intraclass correlation coefficient; model two-way mixed and single measures.

^d^Pearson correlation coefficient.

^e^N/A: not applicable.

^f^Polar heart rate when the measurement of the app is completed and shown.

^g^Polar heart rate immediately when the test finished.

^h^Polar heart rate when starting the measurement with the app.

^i^VO_2_max: maximum oxygen consumption; estimated with the hypothetical arrival at the UKK test finish line considering walking speed and heart rate at P0, P1, and P2, respectively.

**Figure 3:**
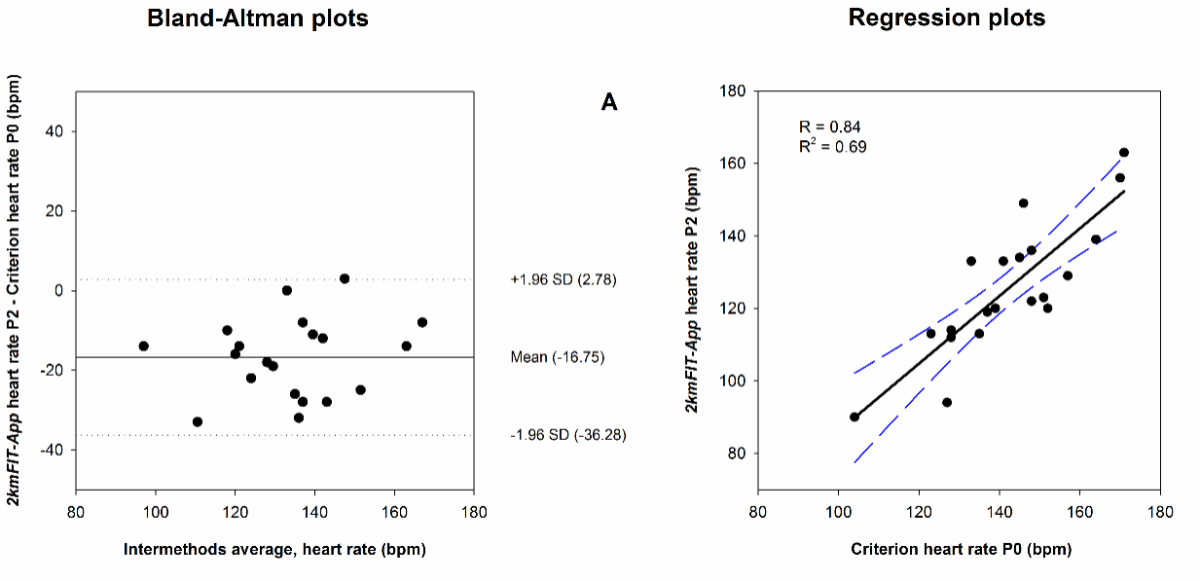
Agreement and regression plots for heart rate as measured by the app (2kmFIT-App, Android version) versus criterion. In the Bland-Altman plot, the central line represents the mean difference (systematic error) and the upper and lower dotted lines represent the 95% limits of agreement (mean difference, SD 1.96 of the differences). Criterion HR P0: polar HR taken immediately when the test finished; R: Pearson correlation coefficient; R2: coefficient of determination; bpm: beats per minute.

**Figure 4:**
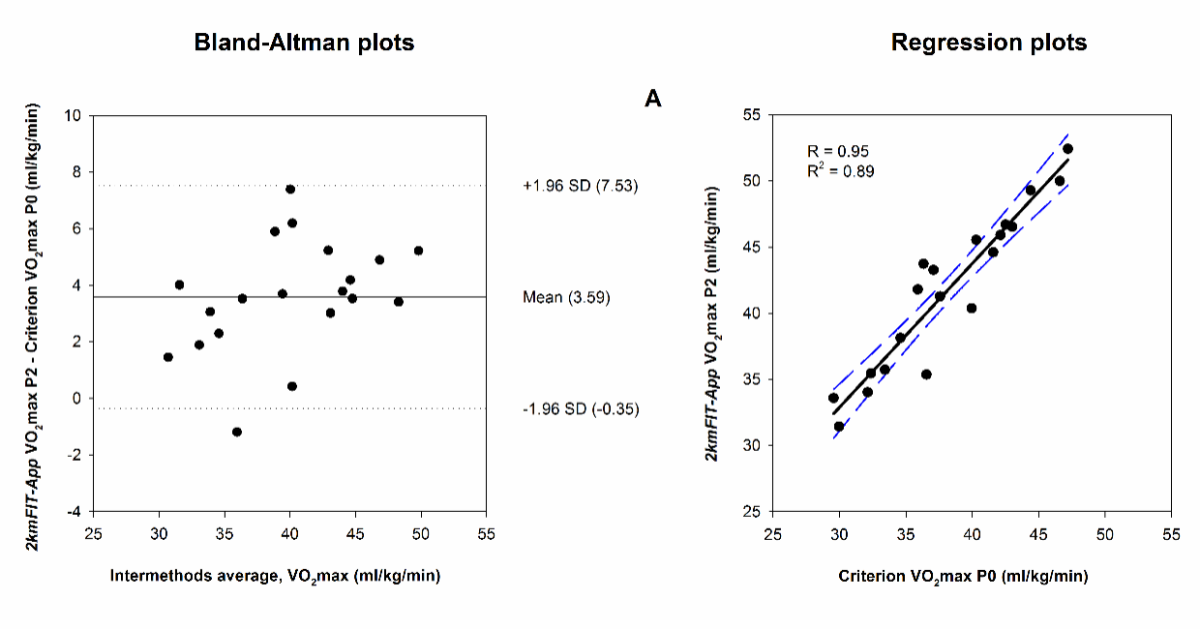
Agreement between the estimated app (2kmFIT-App, Android version) maximal oxygen consumption (VO2max) versus criterion VO2max. The central line represents the mean difference (systematic error) between app and criterion measures. Upper and lower dotted lines represent the 95% limits of agreement (mean difference, SD 1.96 of the differences). R: Pearson correlation coefficient; R2: determination coefficient. Agreement between app VO2max and (A) criterion VO2max P0 (VO2max estimated to the hypothetical arrival at the UKK test finish line considering walking speed and heart rate at P0).

Figures 3 and 4, and Figures S1 and S2 in Multimedia Appendix 1 show the Bland-Altman plots used to evaluate the agreement between the estimated HR and VO_2_max measured through 2kmFIT-App against criterion measures. The MAPE (Table 3) revealed that 2kmFIT-App underestimated distance, time, and HR at P0, and consequently overestimated VO_2_max at P0.

#### Reliability

Table 4 shows the mean differences among app minus test-rest measures within the app for assessing reliability, along with the ICC values. The systematic error was significantly different from zero for distance and pace. There was good reliability as revealed by the ICC for app HR and excellent reliability for app VO_2_max.

**Table 4:** Reliability of 2kmFIT-App (Android version) based on test-retest analysis with the app.

Variable	Test, mean (SD)	Retest, mean (SD)	Difference (retest – test), mean (SD)	*P* value^a^	ICC^b^ (95% CI)	*r^c^*
Distance (meters)	1929.61 (51.47)	1955.60 (34.91)	25.99 (43.21)	.01	0.620 (0.077-0.848)	0.56
Time (minutes)	16.16 (1.93)	16.02 (1.83)	–0.15 (0.94)	.50	0.935 (0.839-0.974)	0.88
Pace (min/km)	8.37 (0.90)	8.20 (0.88)	-0.18 (0.33)	.03	0.957 (0.870-0.984)	0.93
Heart rate P2^d^ (beats/minute)	125.60 (18.32)	121.10 (26.05)	–4.5 (13.44)	.15	0.897 (0.742-0.959)	0.87
VO_2_max P2^e^ (ml/kg/min)	41.75 (6.06)	42.67 (6.18)	0.92 (3.04)	.19	0.932 (0.830-0.973)	0.88

^a^One-sample *t* test.

^b^ICC: intraclass correlation coefficient.

^c^*r:* Pearson correlation coefficient.

^d^Heart rate P2: Polar heart rate when the measurement of the app is completed and shown.

^e^VO_2_max P2: maximum oxygen consumption considering polar heart rate when the measurement of the app is completed.

## Discussion

### Principal Findings

The objective of this study was to analyze the validity and reliability of a smartphone app (2kmFIT-App) for measuring distance, HR, and CRF using Android and iOS mobile operating systems. 2kmFIT-App was demonstrated to be valid and reliable with the Android mobile operating system for measuring CRF, HR, and distance in comparison with CRF estimated by the UKK test, HR measured by a standard Polar HR monitor, and distance measured with a measuring wheel, respectively. First, our validity analysis revealed that 2kmFIT-App (Android, version 2) underestimated distance (–70.40 meters) and HR (–1.95 beats/minute), and overestimated VO_2_max (3.59 ml/min/kg) with an ICC higher than 0.73 for all variables. Second, test-retest reliability showed that 2kmFIT-App (Android, version 2) overestimated distance (25.99 meters) and VO_2_max (0.92 ml/min/kg), but underestimated HR (–4.5 beats/minute). Third, the iOS version of 2kmFIT-App did not obtain accurate measures of HR at medium exercise intensity, thereby its field-test validation and reliability were not further investigated. Collectively, our investigation highlights the potential of 2kmFIT-App as a new and portable device for safely measuring CRF with a low margin of error in the Android mobile operating system.

### In-Laboratory Validation (Phase 1)

One of the major challenges of this investigation was the validity of 2kmFIT-App iOS versions to measure HR. None of the iOS versions we developed achieved precise HR measurements at medium exercise intensities. This lack of accuracy was also found when we tested other commercially available iOS-based HR apps (see Multimedia Appendix 1). This finding concurs with the study of Bouts et al [21] who did not find strong correlations between an electrocardiogram and two iOS-based HR apps (Instant Heart Rate: HR monitor; Runtastic Heart Rate Monitor). More significant bias in HR measurement was found at medium exercise intensities. Although identifying the technological reason for this error is complex, we can speculate a justification for this outcome. The iPhone 6 and later models include a hybrid infrared radiation filter. This filter is designed to reflect or block infrared wavelengths and is usually used to enhance poor lighting conditions. In resting conditions, the difference between oxygenated (red color) and deoxygenated (blue color) blood is low; however, during medium or higher exercise intensities, the contrast becomes higher. Therefore, a possible hypothesis is that the hybrid infrared filter preprocesses the finger image captured (diminishing the contrast between oxygenated and deoxygenated blood), which increases noise in the measurement of HR. Specifically, it could be assumed that the more variability in the blood volume, which occurs at higher frequencies of HR, the greater the measurement error will be.

### Field Test Validation and Reliability (Phase 2)

Our results revealed high levels of agreement between 2kmFIT-App and criterion references, with an ICC ranging from 0.73 to 0.97. Although ICC and *r* values suggested good validity, we also found a systematic error. The criterion-related validity analyses suggested that the app measure of distance provided lower values than the criterion distance, which could be interpreted as a slight underestimation. Specifically, 2kmFIT-App underestimated the distance by 70.40 meters, demonstrating a <4% MAPE from the reference (2 km). Similar to this finding, Benson et al [12] obtained an underestimation of 80 meters (trial 1) and 49 meters (trial 2) with the Motion X GPSTM app against sport-specific global GPS with a criterion distance of 2.4 km. The systematic error between repeated measures (reliability) taken through 2kmFIT-App demonstrated a relatively low error in the distance (25.99 meters). Of note, our investigation was performed in an open-air area (on an athletic track); however, some aspects such as the relatively dense environment (eg, tall buildings, dense vegetation, urban canyons), the manner of carrying the smartphone (pocket or arm), and walking in a straight line or making circles might influence satellite fixing, and therefore precision in distance measurement [22]. Despite these variables, the interest in using apps as stand-alone physical activity monitors is increasing [23]; thereby, our app has proven accuracy in track distance, indicating its validity for determining the walked distance.

2kmFIT-App showed a high degree of validity for measuring HR against Polar RS300X at the end of the UKK test. The systematic error between the app HR and criterion HR at P2 was –1.95 beats/minute, indicating an underestimation of the app with a MAPE <2%. The accuracy of 2kmFIT-App was equivalent regardless of the HR at which the UKK test was finished. Moreover, it is worth mentioning that absolute differences over 20 beats/minute were not observed in any measurement, and only 14.63% (6 measures) reached a difference of up to 10 beats/minute. These results do not concur with those of Coppetti et al [24] who found more than 20% absolute differences at over 20 beats/minutes using some commercially available apps. Furthermore, in contrast to our data, Coppetti and colleagues showed an overestimation for the Instant Heart Rate app (4.52 beats/minute) and Heart Fitness app (1.96 beats/minute) measured at resting conditions. Nevertheless, our results are in line with those of Yan et al [25] who found slight underestimation by the Cardiio smartphone app at different exercise intensities, thereby confirming our findings.

The reliability results (retest minus test) indicated the good reliability of 2kmFIT-App with an ICC of 0.89. Of note, this study was conducted under uncontrolled light and temperature conditions, which might have influenced the absorbed and reflected light by the blood and finger tissues, and in turn increased the measurement error [26]. Thus, using our app in relatively controlled light and temperature environments could achieve more valid results. Although 2kmFIT-App was proven to be a portable and cost-effective tool for monitoring HR in resting and postexercise conditions, such influencing factors should be considered when using the app.

The most salient finding of this study is that the Bland-Altman graph showed valuable information about the high level of agreement for app VO_2_max at P2 against VO_2_max estimated by the UKK test, as revealed by the ICC (0.94) and MAPE (4.93%). Furthermore, the accuracy of the app VO_2_max at P2 was the same regardless of the VO_2_max obtained at the end of the test. In addition, the criterion-related validity analyses suggested that app VO_2_max at P2 provides higher values than the criterion, which was interpreted as an overestimation. According to the UKK test guide, walking time is the most important factor affecting the results of the test. In this sense, 2kmFIT-App underestimated the time by an average of 0.58 minutes, which provides one possible explanation for the overestimation. Another important aspect that likely contributed to the measurement error of app VO_2_max is HR. 2kmFIT-App took an average of 25.85 seconds to measure HR from the end of the test (time at P0 – time at P2), and during that time the HR fell 16.18 beats/minute on average with 59.46% variation (between participants). Thus, the time needed for measuring HR through 2kmFIT-App is the major drawback to be recognized in our investigation. Nevertheless, this limitation could be improved with two actions: decreasing the measurement time of HR by 2kmFIT-App (ie, improving smartphone technology) and applying a correction factor to the equation for estimating app VO_2_max. In this context, the equation VO_2_max corrected=2.24 + 0.89 × X can be used to solve the overestimation of 2kmFIT-App in determining the CRF, in which X is the estimated VO_2_max when using the criterion methods recommended in the original UKK test.

Moreover, reliability analyses revealed excellent agreement (ICC=0.93) within trials (retest minus test) of app VO_2_max. These results suggest the suitability of 2kmFIT-App for monitoring changes in intervention studies with a smaller margin of error, in which precise measurements are needed. Similarly, Brooks et al [27] demonstrated the reliability of the SA-6MWT app to measure exertional capacity using the 6-minute walk test. However, 2kmFIT-App is the only app that is currently able to estimate VO_2_max considering a single physiological measure (HR).

There is no doubt that apps have great potential in clinical and research settings [28]; however, they may not always have an acceptable margin of error. Therefore, researchers, clinicians, and sports specialists should demand scientific validation of apps. In this context, the risk of CRF testing should also be considered, especially in maximal tests. Thus, an app using a submaximal test to estimate CRF is a safer choice, especially when testing is not supervised. 2kmFIT-App is the first validated app for this purpose that is capable of being self-administered and to remotely monitor CRF, making it suitable for most people. Although 2kmFIT-App is suitable for the general population, it can be an especially powerful tool for the prevention and management of cardiovascular disease risk factors [29].

### Limitations and Strengths

A limitation of our study is the time required by 2kmFIT-App to calculate HR immediately at the end of the 2-km walk*.* This fact leads to a decrease in HR (recovery) and results in a slight overestimation of VO_2_max. Moreover, there were some occasional technical issues that should be recognized, such as app freezing or reduced blood circulation in the fingers when a colder temperature could influence data collection. In addition, as a common feature of device-based experiments, the participants were aware of the device worn and the study design, and therefore were not blinded. Additionally, 2kmFIT-App was tested with specific Android hardware, whereas other smartphones with different hardware but the same operating system might potentially present different results. The gold-standard methods to measure HR and VO_2_max were not used in this investigation, since our goal was to test whether we could translate the original UKK test protocol to a smartphone version, and therefore we followed the instructions of the original protocol. The validity of the UKK test to estimate VO_2_max against gas analyzed in laboratory conditions has been proven elsewhere [11,18,30,31]. However, to our knowledge, 2kmFIT-App is the first native app capable of estimating CRF, including a physiological measure (HR), after exercise using PPG technology. The high reliability shown by 2kmFIT-App to estimate CRF, HR, and distance should be recognized as a strength. The possibility of self-administering this test anywhere in the world and at any time makes 2kmFIT-App a powerful tool for public health.

### Conclusion

The results of this study provide important messages for sports specialists and health care professionals. 2kmFIT-App is a new and scientifically validated tool that is capable of objectively and remotely estimating CRF, HR, and distance with a low margin of error in the Android, but not in the iOS, mobile operating system. Given the high reliability achieved, 2kmFIT-App can be used for measuring and monitoring changes precisely with an Android phone. The utility of this app would not only be for the scientific field but also for the millions of people who currently perform physical exercise at a recreational level (not professionally) and want to track their level of CRF.

## Appendix

Multimedia Appendix 1 In-lab validation methods and results, detailed description of 2kmFIT-App structure and content, heart rate protocol, and supplementary results (Figures S1 and S2; Tables S1-S6).

## Abbreviations

CRF: cardiorespiratory fitness
HR: heart rate
ICC: intraclass correlation coefficient
MAPE: mean absolute percentage error
PPG: photoplethysmography
UKK test: 2-km walk test
VO2max: maximal oxygen consumption
