# Supplementary material for: Smartphone App (2kmFIT-App) for Measuring Cardiorespiratory Fitness: Validity and Reliability Study
Source: JMIR Mhealth Uhealth. 2021 Jan 8;9(1):e14864. doi: 10.2196/14864 (PMC7822719; doi:10.2196/14864)
Supplement: Multimedia Appendix 1 [file mhealth_v9i1e14864_app1.docx]

**SUPPLEMENTARY MATERIAL 1**

This supplementary material document provides detailed information concerning the methods and results sections of the manuscript.

**CONTENTS**

[In-Laboratory validation methods (Phase 1) 2](#_Toc521064079)

[2kmFIT-App structure and content 3](#_Toc521064080)

[In-Laboratory validation results (Phase 1) 4](#_Toc521064081)

[Heart rate protocol 5](#_Toc521064082)

[Figure S1. Agreement between the estimated app heart rate (HR) versus criterion HR at two different times (*2kmFIT App*. Android version). 6](#_Toc521064083)

[Figure S2. Agreement between the estimated app VO2max versus criterion VO2max at two different times (*2kmFIT App*. Android version). 7](#_Toc521064084)

[Table S1. Heart rate trials with the *2kmFIT-App* (Android version). 8](#_Toc521064085)

[Table S2. Heart rate trials with the *2kmFIT-App* (iOS version). 9](#_Toc521064086)

[Table S3. Heart rate trials with the Instant Heart Rate: HR monitor. 11](#_Toc521064087)

[Table S4. Heart rate trials with the Runtastic Heart Rate. 12](#_Toc521064088)

[Table S5. Heart rate trials with the Cardiio: Heart Rate Monitor. 13](#_Toc521064089)

[Table S6. Heart rate trials with the HRV4Training. 14](#_Toc521064090)

#

# In-Laboratory validation methods (Phase 1)

During this preliminary validation, different heart rate (HR) measurements with the *2kmFIT-App* were taken with the Android (version 1 and 2) and iOS (version 1. 2 and 3)*,* at two different exercise intensities (rest and moderate intensity). The data from the HR measurement of the app was compared with the RS300X HR monitor from Polar (Kempele, Finland). The HR measurements of the Android version 2 of the *2kmFIT-App* were accurate at both intensities whereas the measurements with the iOS were accurately only at rest (version 1). For this reason, the *2kmFIT-App* Android version 2 was used for testing with participants in the field test validation phase. To note, none of the iOS versions of the *2kmFIT-App* were included in the second phase (field test validation phase 1).

In this preliminary phase, four commercially iOS-based HR apps, stored in the App Store market, were tested at rest and moderate exercise intensity (Instant Heart Rate: HR monitor; Runtastic Heart Rate Monitor; Cardiio: Heart Rate Monitor; HRV4Training). The objective of this trial was to test whether other commercially iOS apps obtain a more valid measure of HR using PPG (photoplethysmographic) imaging than the one obtained with the *2kmFIT App*. The first three apps were selected because they were the top 3 ranked apps in the App Store market for measuring HR. The HRV4Training was selected for being scientifically validated for measuring HR variability.

# 2kmFIT-App structure and content

The *2kmFIT-App* has three main screens. The first screen that pops up when open the app is to start the test (Figure 1A). The second screen allows visual identification of time, distance, and pace while the test is ongoing and graphically show the route by means of GPS (Figure 1B). The last screen enables users to measure HR anytime (Figure 1C). The app has a section where the users can introduce their demographic data to calculate CRF. Additionally, the 2kmFIT-App permits users to share their results through main social networks. The performance of the app to measure CRF is the following: when the users tap the “start test” button (Figure 1A), it appears the screen illustrated in Figure 1B, which shows the distance tracked, time pace and the route on the map during the test. At the end of the test (a final beep is emitted) and the app pop up the third screen (Figure 1C) for HR measurement, then the participant places the finger in the camera and after some seconds of measurement, it appears on the screen the outcomes of the test (Figure 1D).


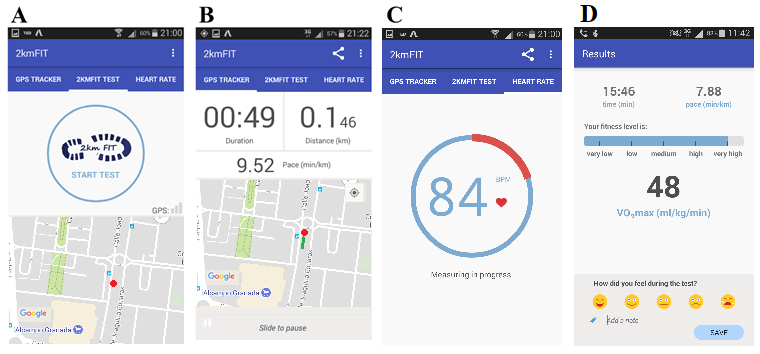


Figure 1. Main screens of the *2kmFIT-App* Android version.

#

# In-Laboratory validation results (Phase 1)

The Android version 2 of the *2kmFIT-App* obtained an acceptable margin error at rest (mean absolute error: 1.20 bpm) and at medium exercise intensity (mean absolute error: 1.10 bpm) in HR measurements. The iOS versions of the *2kmFIT-App* showed accuracy in HR measurements at rest conditions (mean absolute error: 2.6 bpm) whereas none of the versions was precise at medium exercise intensity (mean absolute error range: 11.7 to 53.7 bpm). HRVTraining showed poor validity at rest (mean absolute error: 15.80 bpm) whilst the other three commercially apps achieved better results at rest (mean absolute error range: 1.60 to 3.80 bpm). Instant Heart Rate: HR monitor and Runtastic Heart Rate Monitor showed poor validity in HR measurement at medium exercise intensity (mean absolute error: 16.20 and 46.20 bpm. respectively). The HR measurements at medium exercise intensity with Cardiio: Heart Rate Monitor and HRV4Training were not feasible.

#

# Heart rate protocol

The protocol followed to test heart rate with the different apps was the following:

1. All measurements were taken standing.

2. Burpees were utilized to reach medium intensity.

3. iPhone 6s plus (iOS version 11.3) or Samsung Galaxy SIII Neo (Android 4.4.2) was used for testing the apps.

4. Pulsometer 1: Polar heart rate when starting heart rate measurement with the app.

5. Pulsometer 2: Polar heart rate at the end of heart rate measurement with the app.

6. App: heart rate when the app finished the measurement.





**Figure S1**. Agreement between the estimated app heart rate (HR) versus criterion HR at two different times (2kmFIT App. Android version). Agreement between app HR and (B) criterion HR P1 (Polar HR when starting HR measurement with the app), and (C) criterion HR P2 (Polar HR at the end of HR measurement with the app). R: Pearson correlation coefficient. R^2^: determination coefficient. Central line represents the mean difference (systematic error) between app and criterion measures. Upper and lower dotted lines represent the 95% limits of agreement (mean difference ± 1.96 of the differences).

**

**

**Figure S2**. Agreement between the estimated app VO2max versus criterion VO2max at two different times (2kmFIT App. Android version). Agreement between the estimated app VO_2_max (*2kmFIT App*. Android version) versus criterion VO_2_max in two different times. Agreement between app VO_2_max and: (B) criterion VO_2_max P1 (equal that previous VO_2_max but considering HR P1), (C) and criterion VO_2_max P2 (equal that previous VO_2_max but considering HR P2). Central line represents the mean difference (systematic error) between app and criterion measures. Upper and lower dotted lines represent the 95% limits of agreement (mean difference ± 1.96 of the differences). R: Pearson correlation coefficient. R^2^: determination coefficient.

# Table S1. Heart rate trials with the 2kmFIT-App (Android version).

| Version 2 | *Recovery* | **Rest** |  |  |  | **App Puls 1** | | | **App Puls 2** | | |
| --- | --- | --- | --- | --- | --- | --- | --- | --- | --- | --- | --- |
|  |  |  | *Puls 1* | *Puls 2* | *App* | *Dif App Puls 1* | *Mean Puls 1 App* |  | *Dif App Puls 2* | *Mean Puls 2 App* |  |
|  | 3 | 1 | 62 | 59 | 64 | 2 | 63 | *Mean Puls App* | 5 | 61.5 | *Mean Puls 2 App* |
|  | 3 | 2 | 60 | 63 | 65 | 5 | 62.5 | 62.3 | 2 | 64 | 62.2 |
|  | 5 | 3 | 62 | 57 | 58 | 4 | 60 |  | 1 | 57.5 |  |
|  | 0 | 4 | 64 | 64 | 65 | 1 | 64.5 | *Dif Puls App* | 1 | 64.5 | *Dif Puls App* |
|  | 4 | 5 | 61 | 65 | 62 | 1 | 61.5 | 1 | 3 | 63.5 | 1.2 |
|  |  |  |  |  |  |  |  |  |  |  |  |
| Version 1 |  | **Medium intensity** |  |  |  | **App Puls 1** | | | **App Puls 2** | | |
|  |  |  | *Puls 1* | *Puls 2* | *App* | *Dif App Puls 1* | *Mean Puls 1 App* |  | *Dif App Puls 2* | *Mean Puls 2 App* |  |
|  | 9 | 1 | 165 | 156 | 163 | 2 | 164 | Mean Puls App | 7 | 159.5 | Mean Puls App |
|  | 13 | 2 | 162 | 149 | 155 | 7 | 158.5 | 158.15 | 6 | 152 | 154.75 |
|  | 8 | 3 | 158 | 150 | 146 | 12 | 152 |  | 4 | 148 |  |
|  | 1 | 4 | 159 | 158 | 157 | 2 | 158 | Dif Puls App | 1 | 157.5 | Dif Puls App |
|  | 5 | 5 | 159 | 154 | 149 | 10 | 154 | 7.9 | 5 | 151.5 | 1.1 |
|  | 0 | 6 | 162 | 162 | 153 | 9 | 157.5 |  | 9 | 157.5 |  |
|  | 6 | 7 | 163 | 157 | 158 | 5 | 160.5 |  | 1 | 157.5 |  |
|  | 9 | 8 | 167 | 158 | 158 | 9 | 162.5 |  | 0 | 158 |  |
|  | 15 | 9 | 161 | 146 | 146 | 15 | 153.5 |  | 0 | 146 |  |
|  | 2 | 10 | 165 | 163 | 157 | 8 | 161 |  | 6 | 160 |  |
|  |  |  |  |  |  |  |  |  |  |  |  |
| Version 2 |  | **Medium intensity** |  |  |  | **App Puls 1** | | | **App Puls 2** | | |
|  |  |  | *Puls 1* | *Puls 2* | *App* | *Dif App Puls 1* | *Mean Puls 1 App* |  | *Dif App Puls 2* | *Mean Puls 2 App* |  |
|  | 11 | 1 | 131 | 120 | 125 | 6 | 128 | Mean Puls App | 5 | 122.5 | Mean Puls App |
|  | 7 | 2 | 131 | 124 | 125 | 6 | 128 | 130.8 | 1 | 124.5 | 128.65 |
|  | 6 | 3 | 137 | 131 | 137 | 0 | 137 |  | 6 | 134 |  |
|  | 4 | 4 | 131 | 127 | 129 | 2 | 130 | Dif Puls App | 2 | 128 | Dif Puls App |
|  | 1 | 5 | 132 | 133 | 130 | 2 | 131 | 3.2 | 3 | 131.5 | 1.1 |
|  | 0 | 6 | 130 | 130 | 132 | 2 | 131 |  | 2 | 131 |  |
|  | 3 | 7 | 138 | 135 | 135 | 3 | 136.5 |  | 0 | 135 |  |
|  | 1 | 8 | 136 | 135 | 132 | 4 | 134 |  | 3 | 133.5 |  |
|  | 6 | 9 | 127 | 121 | 119 | 8 | 123 |  | 2 | 120 |  |
|  | 6 | 10 | 131 | 125 | 128 | 3 | 129.5 |  | 3 | 126.5 |  |

*Puls 1: Pulsometer 1; Puls 2: Pulsometer 2; Recovery: Puls 2 minus Puls 1; Dif App Puls 1 was calculated as App minus Puls 1; Dif App Puls 2 was calculated as App minus Puls 2.

# Table S2. Heart rate trials with the 2kmFIT-App (iOS version).

| Version 3 | *Recovery* | **Rest** |  |  |  | **App Puls 1** | | | **App Puls 2** | | |
| --- | --- | --- | --- | --- | --- | --- | --- | --- | --- | --- | --- |
|  |  |  | *Puls 1* | *Puls 2* | *App* | *Dif App Puls 1* | *Mean Puls 1 App* |  | *Dif App Puls 2* | *Mean Puls 2 App* |  |
|  | 3 | 1 | 62 | 59 | 64 | 2 | 63 | *Mean Puls App* | 5 | 61.5 | *Mean Puls 2 App* |
|  | 3 | 2 | 60 | 63 | 67 | 7 | 63.5 | 63 | 4 | 65 | 62.9 |
|  | 5 | 3 | 62 | 57 | 58 | 4 | 60 |  | 1 | 57.5 |  |
|  | 0 | 4 | 64 | 64 | 70 | 6 | 67 | *Dif Puls App* | 6 | 67 | *Dif Puls App* |
|  | 4 | 5 | 61 | 65 | 62 | 1 | 61.5 | 2.4 | 3 | 63.5 | 2.6 |
|  |  |  |  |  |  |  |  |  |  |  |  |
| Version 1 |  | **Medium intensity** |  |  |  | **App Puls 1** | | | **App Puls 2** | | |
|  |  |  | *Puls 1* | *Puls 2* | *App* | *Dif App Puls 1* | *Mean Puls 1 App* |  | *Dif App Puls 2* | *Mean Puls 2 App* |  |
|  | 15 | 1 | 130 | 115 | 72 | 58 | 101 | Mean Puls App | 43 | 93.5 | Mean Puls App |
|  | 15 | 2 | 120 | 105 | 86 | 34 | 103 | 102 | 19 | 95.5 | 95.55 |
|  | 21 | 3 | 132 | 111 | 62 | 70 | 97 |  | 49 | 86.5 |  |
|  | 13 | 4 | 124 | 111 | 68 | 56 | 96 | Dif Puls App | 43 | 89.5 | Dif Puls App |
|  | 15 | 5 | 132 | 117 | 64 | 68 | 98 | 66.6 | 53 | 90.5 | 53.7 |
|  | 10 | 6 | 140 | 130 | 63 | 77 | 101.5 |  | 67 | 96.5 |  |
|  | 5 | 7 | 145 | 140 | 60 | 85 | 102.5 |  | 80 | 100 |  |
|  | 9 | 8 | 143 | 134 | 66 | 77 | 104.5 |  | 68 | 100 |  |
|  | 17 | 9 | 142 | 125 | 75 | 67 | 108.5 |  | 50 | 100 |  |
|  | 9 | 10 | 145 | 136 | 71 | 74 | 108 |  | 65 | 103.5 |  |
|  |  |  |  |  |  |  |  |  |  |  |  |
| Version 2 |  | **Medium intensity** |  |  |  | **App Puls 1** | | | **App Puls 2** | | |
|  |  |  | *Puls 1* | *Puls 2* | *App* | *Dif App Puls 1* | *Mean Puls 1 App* |  | *Dif App Puls 2* | *Mean Puls 2 App* |  |
|  | 3 | 1 | 161 | 164 | 127 | 34 | 144 | Mean Puls App | 37 | 145.5 | Mean Puls App |
|  | 2 | 2 | 159 | 161 | 143 | 16 | 151 | 146.7 | 18 | 152 | 148.25 |
|  | 2 | 3 | 161 | 163 | 149 | 12 | 155 |  | 14 | 156 |  |
|  | 3 | 4 | 160 | 163 | 124 | 36 | 142 | Dif Puls App | 39 | 143.5 | Dif Puls App |
|  | 5 | 5 | 159 | 164 | 145 | 14 | 152 | 26.6 | 19 | 154.5 | 29.7 |
|  | 3 | 6 | 162 | 165 | 136 | 26 | 149 |  | 29 | 150.5 |  |
|  | 3 | 7 | 159 | 162 | 131 | 28 | 145 |  | 31 | 146.5 |  |
|  | 2 | 8 | 159 | 161 | 130 | 29 | 145 |  | 31 | 145.5 |  |
|  | 4 | 9 | 159 | 163 | 128 | 31 | 144 |  | 35 | 145.5 |  |
|  | 4 | 10 | 161 | 165 | 121 | 40 | 141 |  | 44 | 143 |  |

*Continuation of Table S2*

| Version 3 |  | **Medium intensity** |  |  |  | **App Puls 1** | | | **App Puls 2** | | |
| --- | --- | --- | --- | --- | --- | --- | --- | --- | --- | --- | --- |
|  |  |  | *Puls 1* | *Puls 2* | *App* | *Dif App Puls 1* | *Mean Puls 1 App* |  | *Dif App Puls 2* | *Mean Puls 2 App* |  |
|  | 3 | 1 | 161 | 164 | 164 | 3 | 163 | Mean Puls App | 0 | 164 | Mean Puls App |
|  | 3 | 2 | 159 | 162 | 149 | 10 | 154 | 157 | 13 | 155.5 | 158.75 |
|  | 3 | 3 | 161 | 164 | 142 | 19 | 151.5 |  | 22 | 153 |  |
|  | 3 | 4 | 163 | 166 | 148 | 15 | 156 | Dif Puls App | 18 | 157 | Dif Puls App |
|  | 2 | 5 | 160 | 162 | 156 | 4 | 158 | 8.2 | 6 | 159 | 11.7 |
|  | 5 | 6 | 160 | 165 | 152 | 8 | 156 |  | 13 | 158.5 |  |
|  | 5 | 7 | 162 | 167 | 152 | 10 | 157 |  | 15 | 159.5 |  |
|  | 4 | 8 | 163 | 167 | 169 | 6 | 166 |  | 2 | 168 |  |
|  | 2 | 9 | 162 | 164 | 151 | 11 | 156.5 |  | 13 | 157.5 |  |
|  | 5 | 10 | 160 | 165 | 146 | 14 | 153 |  | 19 | 155.5 |  |
|  | *Puls 1: Pulsometer 1; Puls 2: Pulsometer 2. Recovery: Puls 2 minus Puls 1. Dif App Puls 1 was calculated as App minus Puls 1. Dif App Puls 2 was calculated as App minus Puls 2. | | | | | | | | | | |

# Table S3. Heart rate trials with the [Instant Heart Rate: HR monitor](https://itunes.apple.com/us/app/instant-heart-rate-hr-monitor/id409625068?mt=8).

| *Recovery* | **Rest** |  |  |  | **App Puls 1** | | | **App Puls 2** | | |
| --- | --- | --- | --- | --- | --- | --- | --- | --- | --- | --- |
|  |  | *Puls 1* | *Puls 2* | *App* | *Dif App Puls 1* | *Mean Puls 1 App* |  | *Dif App Puls 2* | *Mean Puls 2 App* |  |
| 1 | 1 | 57 | 58 | 57 | 0 | 57 | *Mean Puls App* | 1 | 57.5 | *Mean Puls 2 App* |
| 6 | 2 | 64 | 58 | 58 | 6 | 61 | 58.8 | 0 | 58 | 58.1 |
| 0 | 3 | 62 | 62 | 58 | 4 | 60 |  | 4 | 60 |  |
| 2 | 4 | 60 | 58 | 58 | 2 | 59 | *Dif Puls App* | 0 | 58 | *Dif Puls App* |
| 0 | 5 | 57 | 57 | 57 | 0 | 57 | 2.4 | 0 | 57 | 1 |
|  |  |  |  |  |  |  |  |  |  |  |
|  | **Medium intensity** |  |  |  | **App Puls 1** | | | **App Puls 2** | | |
|  |  | *Puls 1* | *Puls 2* | *App* | *Dif App Puls 1* | *Mean Puls 1 App* |  | *Dif App Puls 2* | *Mean Puls 2 App* |  |
| 23 | 1 | 132 | 109 | 129 | 3 | 130.5 | Mean Puls App | 20 | 119 | Mean Puls App |
| 28 | 2 | 134 | 106 | 110 | 24 | 122 | 128 | 4 | 108 | 114.7 |
| 30 | 3 | 137 | 107 | 140 | 3 | 138.5 |  | 33 | 123.5 |  |
| 36 | 4 | 132 | 96 | 111 | 21 | 121.5 | Dif Puls App | 15 | 103.5 | Dif Puls App |
| 16 | 5 | 131 | 115 | 124 | 7 | 127.5 | 10.4 | 9 | 119.5 | 16.2 |

*Puls 1: Pulsometer 1; Puls 2: Pulsometer 2. Recovery: Puls 2 minus Puls 1. Dif App Puls 1 was calculated as App minus Puls 1. Dif App Puls 2 was calculated as App minus Puls 2.

#

# Table S4. Heart rate trials with the [Runtastic Heart Rate](https://itunes.apple.com/us/app/runtastic-heart-rate-pro/id586956623?mt=8).

| *Recovery* | **Rest** |  |  |  | **App Puls 1** | | | **App Puls 2** | | |
| --- | --- | --- | --- | --- | --- | --- | --- | --- | --- | --- |
|  |  | *Puls 1* | *Puls 2* | *App* | *Dif App Puls 1* | *Mean Puls 1 App* |  | *Dif App Puls 2* | *Mean Puls 2 App* |  |
| 3 | 1 | 63 | 60 | 57 | 6 | 60 | *Mean Puls App* | 3 | 58.5 | *Mean Puls 2 App* |
| 5 | 2 | 62 | 57 | 53 | 9 | 57.5 | 58.4 | 4 | 55 | 56.5 |
| 4 | 3 | 60 | 56 | 53 | 7 | 56.5 |  | 3 | 54.5 |  |
| 8 | 4 | 67 | 59 | 57 | 10 | 62 | *Dif Puls App* | 2 | 58 | *Dif Puls App* |
| 1 | 5 | 59 | 60 | 53 | 6 | 56 | 7.6 | 7 | 56.5 | 3.8 |
|  |  |  |  |  |  |  |  |  |  |  |
|  | **Medium intensity** |  |  |  | **App Puls 1** | | | **App Puls 2** | | |
|  |  | *Puls 1* | *Puls 2* | *App* | *Dif App Puls 1* | *Mean Puls 1 App* |  | *Dif App Puls 2* | *Mean Puls 2 App* |  |
| 22 | 1 | 129 | 107 | 56 | 73 | 92.5 | Mean Puls App | 51 | 81.5 | Mean Puls App |
| 52 | 2 | 132 | 80 | 55 | 77 | 93.5 | 95.8 | 25 | 67.5 | 82.7 |
| 16 | 3 | 127 | 111 | 56 | 71 | 91.5 |  | 55 | 83.5 |  |
| 23 | 4 | 137 | 114 | 48 | 89 | 92.5 | Dif Puls App | 66 | 81 | Dif Puls App |
| 18 | 5 | 135 | 117 | 83 | 52 | 109 | 72.4 | 34 | 100 | 46.2 |
| *Puls 1: Pulsometer 1; Puls 2: Pulsometer 2. Recovery: Puls 2 minus Puls 1. Dif App Puls 1 was calculated as App minus Puls 1. Dif App Puls 2 was calculated as App minus Puls 2. | | | | | | | | | | |

#

#

# Table S5. Heart rate trials with the [Cardiio: Heart Rate Monitor](https://itunes.apple.com/us/app/cardiio-heart-rate-monitor/id542891434?mt=8).

| *Recovery* | **Rest** |  |  |  | **App Puls 1** | | | **App Puls 2** | | |
| --- | --- | --- | --- | --- | --- | --- | --- | --- | --- | --- |
|  |  | *Puls 1* | *Puls 2ª* | *App* | *Dif App Puls 1* | *Mean Puls 1 App* |  | *Dif App Puls 2* | *Mean Puls 2 App* |  |
| 2 | 1 | 55 | 57 | 61 | 6 | 58 | *Mean Puls App* | 4 | 59 | *Mean Puls 2 App* |
| 0 | 2 | 54 | 54 | 54 | 0 | 54 | 55.1 | 0 | 54 | 55 |
| 1 | 3 | 55 | 56 | 55 | 0 | 55 |  | 1 | 55.5 |  |
| 1 | 4 | 55 | 54 | 54 | 1 | 54.5 | *Dif Puls App* | 0 | 54 | *Dif Puls App* |
| 3 | 5 | 53 | 50 | 55 | 2 | 54 | 1.4 | 5 | 52.5 | 1.6 |
|  |  |  |  |  |  |  |  |  |  |  |
|  | **Medium intensity*** |  |  |  | **App Puls 1** | | | **App Puls 2** | | |
|  |  | *Puls 1* | *Puls 2* | *App* | *Dif App Puls 1* | *Mean Puls 1 App* |  | *Dif App Puls 2* | *Mean Puls 2 App* |  |
| 0 | 1 |  |  |  | 0 | 0 | Mean Puls App | 0 | 0 | Mean Puls App |
| 0 | 2 |  |  |  | 0 | 0 | 0 | 0 | 0 | 0 |
| 0 | 3 |  |  |  | 0 | 0 |  | 0 | 0 |  |
| 0 | 4 |  |  |  | 0 | 0 | Dif Puls App | 0 | 0 | Dif Puls App |
| 0 | 5 |  |  |  | 0 | 0 | 0 | 0 | 0 | 0 |
| *Puls 1: Pulsometer 1; Puls 2: Pulsometer 2. Recovery: Puls 2 minus Puls 1. Dif App Puls 1 was calculated as App minus Puls 1. Dif App Puls 2 was calculated as App minus Puls 2. | | | | | | | | | | |
|  |  |  |  |  |  |  |  |  |  |  |
|  | *It was impossible to make a measurement at a medium intensity. | | | | | |  |  |  |  |

# Table S6. Heart rate trials with the [HRV4Training](https://www.hrv4training.com/).

| *Recovery* | **Rest** |  |  |  | **App Puls 1** | | | **App Puls 2** | | |
| --- | --- | --- | --- | --- | --- | --- | --- | --- | --- | --- |
|  |  | *Puls 1* | *Puls 2* | *App* | *Dif App Puls 1* | *Mean Puls 1 App* |  | *Dif App Puls 2* | *Mean Puls 2 App* |  |
| 4 | 1 | 53 | 49 | 69 | 16 | 61 | *Mean Puls App* | 20 | 59 | *Mean Puls 2 App* |
| 1 | 2 | 55 | 56 | 65 | 10 | 60 | 61.3 | 9 | 60.5 | 60.3 |
| 1 | 3 | 53 | 52 | 60 | 7 | 56.5 |  | 8 | 56 |  |
| 5 | 4 | 56 | 51 | 72 | 16 | 64 | *Dif Puls App* | 21 | 61.5 | *Dif Puls App* |
| 1 | 5 | 55 | 54 | 75 | 20 | 65 | 13.8 | 21 | 64.5 | 15.8 |
|  |  |  |  |  |  |  |  |  |  |  |
|  | **Medium intensity*** |  |  |  | **App Puls 1** | | | **App Puls 2** | | |
|  |  | *Puls 1* | *Puls 2* | *App* | *Dif App Puls 1* | *Mean Puls 1 App* |  | *Dif App Puls 2* | *Mean Puls 2 App* |  |
| 0 | 1 |  |  |  | 0 | 0 | Mean Puls App | #¡VALOR! | 0 | Mean Puls App |
| 0 | 2 |  |  |  | 0 | 0 | 0 | 0 | 0 | 0 |
| 0 | 3 |  |  |  | 0 | 0 |  | 0 | 0 |  |
| 0 | 4 |  |  |  | 0 | 0 | Dif Puls App | 0 | 0 | Dif Puls App |
| 0 | 5 |  |  |  | 0 | 0 | 0 | 0 | 0 | 0 |
| *Puls 1: Pulsometer 1; Puls 2: Pulsometer 2. Recovery: Puls 2 minus Puls 1. Dif App Puls 1 was calculated as App minus Puls 1. Dif App Puls 2 was calculated as App minus Puls 2. | | | | | | | | | | |
|  |  |  |  |  |  |  |  |  |  |  |
|  | *The app does not allow to estimate the HR in less than a minute. Therefore. no medium intensity trials were taken because HR dropped ± 20 beats per minute during this minute. | | | | | | | | | |
